# Supplementary material for: From sequence to enzyme mechanism using multi-label machine learning
Source: BMC Bioinformatics. 2014 May 19;15:150. doi: 10.1186/1471-2105-15-150 (PMC4229970; doi:10.1186/1471-2105-15-150)
Supplement: Additional file 2 — Java code of ml2db. Additional file ml2db_code.tar.gz contains the Java source code to run the multi-label machine learning experiments and save the results to database. The code’s Javadoc is included. [file 1471-2105-15-150-S2.zip › additional file 2/ml2db/ml2db/doc/mulan/classifier/meta/MultiLabelMetaLearner.html]

MultiLabelMetaLearner


JavaScript is disabled on your browser.


- Overview
- Package
- Class
- Use
- Tree
- Deprecated
- Index
- Help

- Prev Class
- Next Class

- Frames
- No Frames

- All Classes

- Summary:
- Nested |
- Field |
- Constr |
- Method

- Detail:
- Field |
- Constr |
- Method


mulan.classifier.meta

## Class MultiLabelMetaLearner

- java.lang.Object
- - mulan.classifier.MultiLabelLearnerBase
  - - mulan.classifier.meta.MultiLabelMetaLearner

- All Implemented Interfaces:
  :   java.io.Serializable, MultiLabelLearner, weka.core.TechnicalInformationHandler

  Direct Known Subclasses:
  :   ClusteringBased, EnsembleOfSubsetLearners, ExampleBasedFMeasureOptimizer, HMC, HMCNode, HOMER, Meta, MLPTO, OneThreshold, RAkEL, RAkELd, RCut, SCut, SubsetLearner

  ---

    

  ```
  public abstract class MultiLabelMetaLearner
  extends MultiLabelLearnerBase
  ```

  Base class for multi-label learners, which use other multi-label learners

  Version:
  :   $Revision: 1.1 $

  Author:
  :   Grigorios Tsoumakas

  See Also:
  :   Serialized Form

- - ### Constructor Summary

    Constructors

    Constructor and Description

    `MultiLabelMetaLearner(MultiLabelLearner baseLearner)`

    Creates a new instance.
  - ### Method Summary

    Methods

    | Modifier and Type | Method and Description |
    | --- | --- |
    | `MultiLabelLearner` | `getBaseLearner()` Returns the `MultiLabelLearner` which is used internally by the learner. |

    - ### Methods inherited from class mulan.classifier.MultiLabelLearnerBase

      `build, getDebug, getTechnicalInformation, globalInfo, isUpdatable, makeCopy, makePrediction, setDebug`
    - ### Methods inherited from class java.lang.Object

      `equals, getClass, hashCode, notify, notifyAll, toString, wait, wait, wait`

- - ### Constructor Detail


    - #### MultiLabelMetaLearner

      ```
      public MultiLabelMetaLearner(MultiLabelLearner baseLearner)
      ```

      Creates a new instance.

      Parameters:
      :   `baseLearner` - the base multi-label learner which will be used
          internally to handle the data.
  - ### Method Detail


    - #### getBaseLearner

      ```
      public MultiLabelLearner getBaseLearner()
      ```

      Returns the `MultiLabelLearner` which is used internally by the
      learner.

      Returns:
      :   the baseLearner


- Overview
- Package
- Class
- Use
- Tree
- Deprecated
- Index
- Help

- Prev Class
- Next Class

- Frames
- No Frames

- All Classes

- Summary:
- Nested |
- Field |
- Constr |
- Method

- Detail:
- Field |
- Constr |
- Method
